# Celebrating 100 years of insulin with Dr Elizabeth Seaquist

**DOI:** 10.1242/dmm.049351

**Published:** 2021-11-11

**Authors:** Elizabeth Seaquist


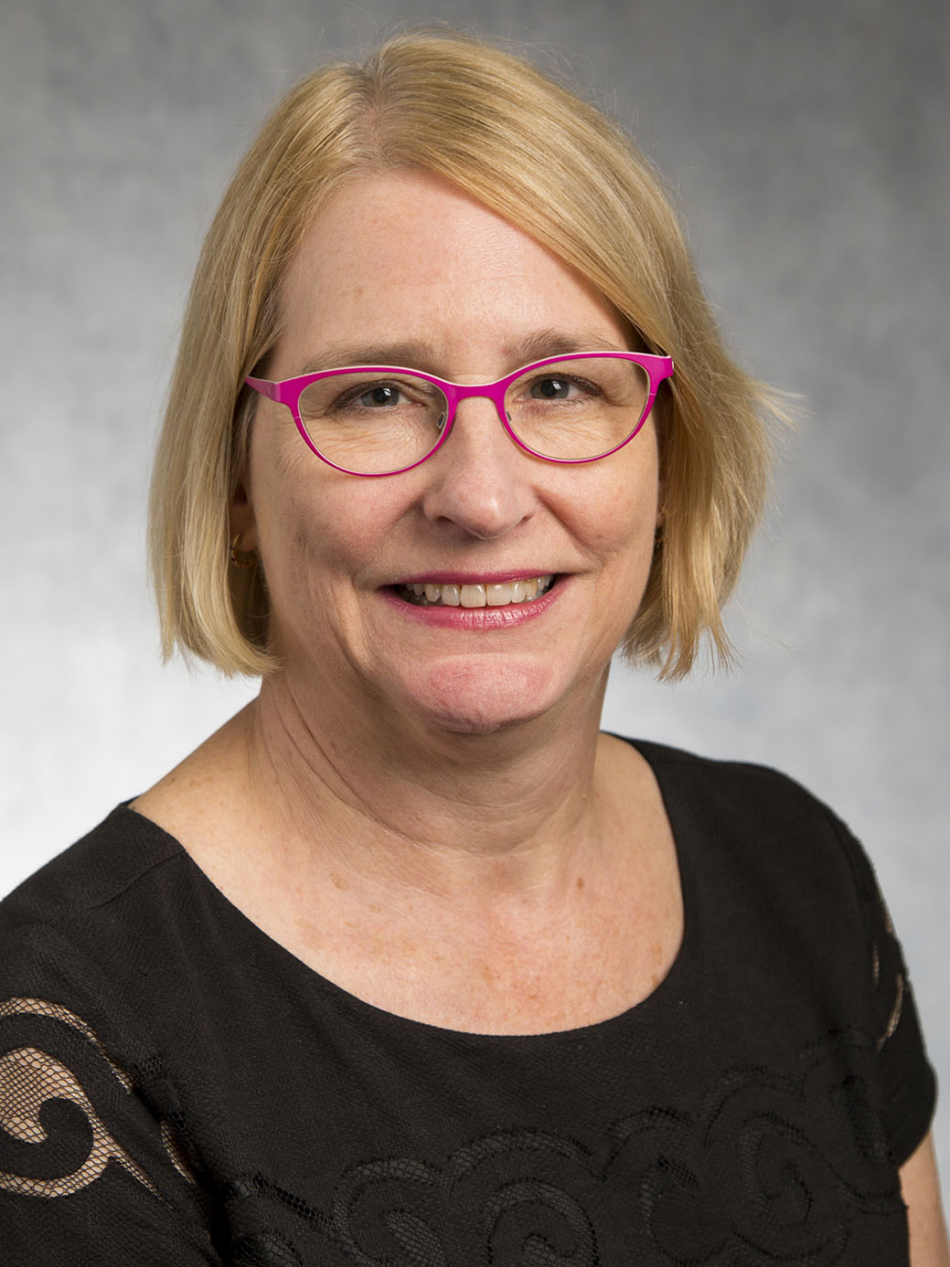


Dr Elizabeth Seaquist has dedicated her career to improving the lives of people with diabetes. She obtained her medical training at the University of Minnesota, where she specialised in endocrinology, diabetes and metabolism. She then pursued research in this speciality and continues to contribute to this field as a professor of medicine at the University of Minnesota and affiliated hospitals, where she also leads several clinical trials in diabetes. She holds numerous leadership roles within these faculties and was appointed as the President for Medicine and Science of the American Diabetes Association in 2014. Her contributions to medicine and research have been recognised with many awards, including the American Diabetes Association's Transformative Woman in Diabetes Award in 2019 and the Mary Jane Kugel Award from the Juvenile Diabetes Research Foundation.

2021 marks the 100-year anniversary of the discovery of insulin by Frederick Banting, Charles Best and J. J. R. Macleod. This remarkable discovery made the treatment of diabetes, a previously lethal condition, possible. However, when diabetes patients administer insulin, there is a risk of hypoglycaemia occurring if blood glucose levels are reduced excessively. Dr Seaquist's research delves into the effects of diabetes complications, in particular hypoglycaemia, on the cardiovascular system and the brain. In this ‘A Model for Life’ interview, she shares her experiences as a clinician and researcher in the field of diabetes and reflects upon the progression of diabetes research over the past 100 years and beyond.

“[…] helping patients figure out how to live their life with diabetes, as opposed to having diabetes run their life, has been an incredibly rewarding career.”

**How did you decide to specialise in endocrinology and, more specifically, in diabetes?**

When I went to medical school, I already knew I was interested in hormonal regulation and physiology. I majored in biology in college, and there I was really interested in how different organs talk to each other, which is exactly what endocrinology is. However, at every step in my training, I fought the urge to narrow my focus, because I really liked medicine broadly and taking care of patients. So, I decided I would train in internal medicine because that lets me treat all adults. I actually wasn't sure I was going to specialise in endocrinology until my second year of residency. I realised if I were to specialise in diabetes, I could keep my focus broad, because diabetes affects every organ system. I have also always been very interested in the impact of behaviour on health, and, certainly, behaviour is a major part of managing diabetes. So, I thought this was a way to keep my broad focus, while at the same time becoming an expert in something. I love endocrinology broadly, but helping patients figure out how to live their life with diabetes, as opposed to having diabetes run their life, has been an incredibly rewarding career.

**Do you think lifestyle coaching should be included in more clinical trials?**

It's fundamental to everything we do in diabetes management. The very first thing you have to consider is lifestyle management, which means people need to understand how the food that they eat affects their blood sugar and their weight. And the same with exercise. It's not complicated, but it's all encompassing. Every decision a person makes throughout the day can affect their blood sugars, and that's overwhelming for people. I was talking with one of my mentees earlier this week, who was studying the impact of diet on type two diabetes, and we're thinking about new grant ideas for her. She made the point that we think about lifestyle modification very early in the course of diabetes, but we fail to conscientiously go back to it over and over again, which we should because it's so fundamental. We need to continue to focus on this, especially as we think about how to individualise therapies for the patient in front of us. People live their lives a certain way based on what they've grown up with, and what they think is normal. Our job as diabetes doctors is to help ensure that, within the context of their life, they can make good choices that will help them manage their diabetes.

**In your experience, what are the advantages and challenges of being active in both the clinic and research?**

I do think it's a tremendous advantage to be a physician who cares for patients with diabetes and a diabetes investigator. I always have the opportunity to think about the newest advances that are coming, which is an advantage to my patients. On the other hand, as a scientist, my best ideas, and the best projects I spend my life studying, are all based on what patients tell me. I've spent my life trying to understand why recurrent hypoglycaemia blunts the counter-regulatory response to future hypoglycaemia – why people who have lots of hypoglycaemic events fail to notice when their blood sugar levels are getting low and then become unconscious. I think it's just a horrendous event, and the reason I care about this is because it happens to my patients. They tell me, “I didn't know my blood sugar levels were getting low and I ended up in a car accident”. We have to figure this out and, to do so, we should spend our time investigating issues that are important to our patients.

The other benefit is that, because I study human subjects, I have patients available to me who may want to participate in my research. But that's also where one of the challenges comes in. As a physician, my focus is on the wellbeing of the patient in front of me; as a scientist, recruiting someone for a trial, I want to be certain someone understands what it means to be in the trial, the risks and benefits and what they have to do. If I request consent, there's always a risk that my patients will agree because they want to please me, or they want to help me out, which makes it challenging to be certain that I'm really getting informed consent. This is one of the things clinical investigators need to think about and address straightforwardly with patients. You need to let them know that you feel this conflict. Fortunately, I have a big team and I don't usually request consent from patients anymore. Although early in my career I did, and it was a challenge. I think you have to be certain that you're making it clear to people what your role is at the point of when you are getting their consent to participate in a study.

**What inspired you to pursue research after doing your medical training?**

I was really inspired to do research even before my medical training. I was fortunate to have an outstanding biology teacher in high school who let me loose in the lab. I added a lot more biology courses than I needed to graduate from high school and a lot of it was independent study. She encouraged me just to ask questions, plan an experiment and carry out these experiments. Then I ended up working in a haematology lab during college, which was really fun. I also went to a college that really encouraged people to do independent research. Then, after college, I spent a year in an obesity lab in New York City. All along the way, I really loved research, but I never thought I would become a researcher. I always thought I was going to be a physician and hoped I could incorporate research. Then I was fortunate that I made choices that allowed me to do further training so that I could become an investigator, which takes a long time. I didn't realise how long it takes to do the training, but I'm glad I did it.

**Your research investigates hypoglycaemia and associated complications in the cardiovascular system and the brain. Which aspect of your research has been most exciting to work on?**

The impact of diabetes on the brain is really interesting to me. I have been fortunate to have collaborators who have really supported my interest in the brain, such as Gulin Oz and Silvia Mangia. I'm not trained as a neurobiologist or a psychiatrist, but in the past 20 years, we have really come to appreciate the interconnectedness of the endocrine system, the brain and the nervous system as a whole. I think there is a lot of crosstalk between those systems regulating overall metabolism and physiology that we didn't initially appreciate. Certainly, when I was in medical school, these systems were investigated separately.

I'm really fascinated by the idea that diabetes, hypoglycaemia and hypoglycaemia-related factors that lead to diabetes complications can actually have an effect on the brain itself, including the neurons and the glial cells. I would love to understand this, and I have spent a lot of time thinking about it. Our tools aren't sophisticated enough yet for us to really study this on a cellular basis in living people. That's what I always want to do – understand what is happening in this living person in front of me and what impact it has. I'm very intrigued by finding out how recurrent hypoglycaemia alters the way neurons talk to each other, making people respond differently. There's some evidence of this, but it's hard to come by in human studies. Hopefully, the next generation will figure it out and I'll be able to read the reports, as an old lady not doing the work anymore.

“I can't imagine having been alive in 1921 and 1922, taking care of patients with diabetes who were dying from the disease. All of a sudden, with the appearance of insulin, people with diabetes were living.”

**As it is 100 years since the discovery of insulin, what do you think has been the most significant discovery in diabetes research since then?**

I can't imagine having been alive in 1921 and 1922, taking care of patients with diabetes who were dying from the disease. All of a sudden, with the appearance of insulin, people with diabetes were living. What an enormous impact that discovery had. Although it was far from perfect, as even Banting and Best recognised that hypoglycaemia was a problem, it was still lifesaving. I'm really impressed with all of the advances that have been made through medicinal chemistry, making insulin easier for people to use. We have new insulins and other therapies for diabetes. I'm very excited about the developments with the ultra-long insulins that are administered once a week, as well as the faster mealtime insulins, and the smart insulins that turn on and turn off depending on what your blood sugar level is. It's a technology that's evolving, and we may have some amazing choices in the next few years.

The other thing that has been earth shaking in clinical practice has been device development. In particular, continuous glucose monitors give patients an opportunity to manage their diabetes that they never had before. If you check your blood sugar four times a day, the levels may go up and down in between each of those readings, which has a huge impact on your life. And so, I do think the miniaturisation of the continuous glucose monitor has really improved management, certainly for people with type one diabetes. But even for people with type two diabetes who don't take insulin, it reinforces lifestyle choices. It really empowers the patient to think about how their own choices can impact their blood sugar levels. I think it's really a transformative technology and having that immediate feedback is amazing.

**Obviously, insulin is an integral part of the treatment of diabetes. But if you look into the future, do you think there’s going to be a time when patients and clinicians can become less reliant on insulin?**

I think for insulin-deficient people, unless we can figure out a way to get their bodies to make insulin itself, we're going to have to rely on pharmacologic therapy for a while. Although we've made progress in type one diabetes, especially understanding the immune nature of it, we haven't figured out a way to restore beta cells once they're gone. There is a lot of effort investigating this, but we still have a long way to go. I do think for people who are able to make some insulin, we will be able to continue non-insulin therapies for longer to manage their blood sugar levels. It really highlights how important and central insulin is. Of course, we have the ability to turn it on quickly, but we also have to figure out how to turn it off more quickly, in a safe way, so that we can act, therapeutically, more like the pancreas does.

**You continue to do a lot of clinical studies. What do you think are the key factors for a successful clinical study or even just a collaborative study in general?**

I've been fortunate to have amazing collaborators and a team that has been together for years. I have a physician assistant by the name of Anjali Kumar, who is incredibly good with operations and wonderful with patients. You really need staff who are dedicated to the task ahead of you, and who work with you day in and day out to make sure things happen.

You also need to ensure that you bring in people who have expertise different from your own. My colleagues Gulin Oz and Silvia Mangia and others are magnetic resonance experts. Although I've used magnetic resonance spectroscopy and imaging in my studies, I am not an expert in these fields. I've learned so much from my collaborators, but I think they've learned a lot from me and my team about diabetes. Having the chance to grow together is really important and I think science is changing in that respect. When I was a junior investigator, there was a lot of focus on supporting your own career development and being independent. But I think science has gotten so complex that a single person can't be responsible for mastering all of it. And that's a challenge because it leaves you reliant upon your colleagues. You must trust that they're being conscientious with the application of their technology and interpretation of data, which requires a strong relationship, where you really are connected as a team. I think that we're still progressing with this, because promotion and tenure at most universities pushes investigators to be independent and successful on their own, but most people will find it much easier to be successful in partnership with others. Also, I think we have to be aware that teams with junior and senior researchers have power dynamics in them, and we need to be certain that we're giving a voice to people who have less experience as well. We have to be intentional about that. My collaborators and I continued to meet weekly online throughout the pandemic, even though our experiments were shut down for a while, so that we could touch base and talk about ideas.

“[…] it's all about helping the mentee use their gifts most successfully for their own satisfaction and for the good of the world.”

**You dedicate much of your efforts to mentoring. You are the principal investigator on the National Institutes of Health Research Training Grant for fellows in endocrinology and diabetes at the University of Minnesota and you've been awarded the Albert Renold Award for Mentoring in 2020. Could you tell us about your mentoring philosophy?**

I've been really fortunate to have good mentees, and I think the key is making sure that, as a mentor, I know and understand the goals of the mentee. The world is not going to be a better place if there are 100 replicas of me. We need much more creativity and innovation than that. And so, I try to identify what someone wants to accomplish in the long term. Where do they want to be in 5 years and what steps do they need to take to get there? Most of the time a mentor knows much better than the mentee what steps need to be taken. We have to track that with a timeline, and my job is to hold the mentee accountable to that timeline. Inevitably, there will be challenges in meeting those goals and we will face barriers, which will give the mentee insights into some of the challenges in becoming a successful investigator. There are a lot of these challenges, including time management, being distracted by clinical duties, or having to prioritise something else over their research. The mentee will learn what they really need to work on. Then I have to be sure that I'm continually going in the direction in which the mentee wants to go, as maybe they'll reconsider things. You have to be open to that possibility because it's all about helping the mentee use their gifts most successfully for their own satisfaction and for the good of the world. That only comes when the person is true to themselves.

**Do you think it's important for young female clinicians and researchers to see women, such as yourself, in leadership roles?**

I do. I really do. I'm old enough that there were very few role models for me to look up to. There were a handful, certainly, but very few and far between. I think it makes a difference to see people that look like you and have had similar choices in life to you, that do the work you want to do. And so, I do think it's really important that we have people of colour and women in these leadership roles. We need a much more diverse scientific and physician workforce, particularly in the United States and in Europe, where these roles are still predominantly held by white men. People always talk about how if we just wait over time, it will eventually change because more women and more people of colour are coming into medical school. Well, they've been saying this for decades. And in fact, it is better. But there's still no proportionality. We've had 50/50 classes in medical school with respect to men and women for a long time and we don't see that equality filtering through to leadership roles. That tells me we need to think about this a little more intentionally. Why are people making choices to leave or not pursue a certain path? We have to make sure that the micro-aggressions, that can be so disheartening over the long haul and dissuade people from going forward, are not allowed to happen anymore. I think most academic institutions are really facing that head on and talking about it now.

**You're obviously very passionate about science and medicine, but is there an alternative career path you would have considered?**

I applied to medical school 2 years in a row. After the second year, it looked like I might not get in and then I ultimately did off a waiting list. But I wasn't going to apply a third time, so I was going to be a special education teacher. I had always been interested in special education and children with special needs. I grew up with a sister who had special needs, and I was very interested in that education. So, I thought I would get a graduate degree in that and pursue it as a career. I'm glad I had the opportunity to go to medical school. I am very passionate about it, but I sometimes wonder if I had gone in the other direction where I would be today. What I've learned about myself is that I like to lead things. So, I probably would be doing something more administrative at this point in my education career. My interest in special education has still remained. Over the years, a number of patients with diabetes have had special needs, and I've really enjoyed participating in their care, because it requires a different way of thinking about things, which has been very challenging, but good.

